# Editorial for Special Issue “Effects of Nanoparticles on Living Organisms 2.0”

**DOI:** 10.3390/cimb47010046

**Published:** 2025-01-13

**Authors:** Yoshitaka Miyamoto

**Affiliations:** 1 Department of Maternal-Fetal Biology, National Research Institute for Child Health and Development, Setagaya-ku, Tokyo 157-8535, Japan; myoshi1230@gmail.com or miyamoto-ys@ncchd.go.jp; Tel.: +81-3-3416-0181; 2 Graduate School of Engineering, Tokyo University of Agriculture and Technology, Koganei, Tokyo 184-8588, Japan; 3 Department of Mechanical Engineering, Institute of Science Tokyo, 12-2-1 Ookayama, Meguro-ku, Tokyo 152-8552, Japan

This Special Issue provides an overview of the “Effects of Nanoparticles on Living Organisms 2.0”. Nanoparticles are used in food, agriculture, drug discovery, and medicine (pharmaceuticals, prevention, and diagnosis) and, above all, the studies reported in this Special Issue demonstrate applications in and potential of nanoparticles in the medical field, but also how they can effectively be applied in other fields. Currently, most nanoparticles in clinical trials [1,2] are used as nanomedicine and drug delivery carriers, and the most common types of nanoparticles are protein-, polymeric-, and lipid-based nanoparticles, while others include metallic-based nanoparticles and nanocrystals. Nanoparticles are also applied in medicine and related technologies, such as biomedical imaging [3], cell sorting and targeting [4], and gene transfer [5], which are also covered in this Special Issue. For this Special Issue, a comprehensive review process was conducted for the received submissions, and seven high-quality works were accepted for publication.

There are five original articles on nanoparticles in this Special Issue. Varlı HS et al. [6] studied the reprogramming from human fibroblasts to induced pluripotent stem (iPS) cells using octadecylamine-based solid lipid nanoparticles. Riet K et al. [7] encapsulated *Euphorbia milii* DCM root extract in thermosensitive liposomes and studied its effects in prostate cancer cells. Babonaitė M et al. [8] investigated the DNA-damaging properties of PVP-coated silver, silica, aluminum oxide, and gold nanoparticles in human peripheral blood mononuclear cells. Tyavambiza C et al. [9] synthesized silver nanoparticles (Cotyledon-AgNPs) using an extract of Cotyledon orbiculata and investigated their safety for use in wound healing. Morozova O et al. [10] evaluated protein nanoparticles’ stability in biological fluids and their distribution in the organs of animals after intranasal and oral administration. There are also two review articles on nanoparticles in this Special Issue. Matalqah S et al. [11] summarize an overview of hyaluronic acid-based nanopharmaceuticals, and Lukhele BS et al. [12] summarize plant material-loaded vesicular drug delivery systems for pulmonary diseases.

In conclusion, we have selected for publication manuscripts that evaluate the “Effects of Nanoparticles on Living Organisms 2.0” for medical technology and application.

Dr. Yoshitaka Miyamoto would like to thank the Editor-in-Chief, Prof. Dr. Madhav Bhatia, as well as the editorial team and the reviewers of *Current Issues in Molecular Biology*, who helped us on the journey to publishing this Special Issue.

In this Special Issue, many papers are in the medical and material fields, including nanoparticles used as pharmaceuticals, drug delivery systems, and medical technology. Therefore, we look forward to receiving submissions from more diverse fields in the next Special Issue, “Effects of Nanoparticles on Living Organisms, 3rd Edition”.

## References

[1] Bobo D., Robinson K.J., Islam J., Thurecht K.J., Corrie S.R. (2016). Nanoparticle-Based Medicines: A Review of FDA-Approved Materials and Clinical Trials to Date. Pharm. Res..

[2] Namiot E.D., Sokolov A.V., Chubarev V.N., Tarasov V.V., Schiöth H.B. (2023). Nanoparticles in Clinical Trials: Analysis of Clinical Trials, FDA Approvals and Use for COVID-19 Vaccines. Int. J. Mol. Sci..

[3] Han X., Xu K., Taratula O., Farsad K. (2019). Applications of nanoparticles in biomedical imaging. Nanoscale.

[4] Cheng Q., Wei T., Farbiak L., Johnson L.T., Dilliard S.A., Siegwart D.J. (2020). Selective organ targeting (SORT) nanoparticles for tissue-specific mRNA delivery and CRISPR-Cas gene editing. Nat. Nanotechnol..

[5] Kavanagh E.W., Green J.J. (2022). Toward Gene Transfer Nanoparticles as Therapeutics. Adv. Healthc. Mater..

[6] Varlı H.S., Yıldırım M.A., Kızılbey K., Türkoğlu N. (2024). Gene Delivery via Octadecylamine-Based Nanoparticles for iPSC Generation from CCD1072-SK Fibroblast Cells. Curr. Issues Mol. Biol..

[7] Riet K., Adegoke A., Mashele S., Sekhoacha M. (2024). Effective Use of Euphorbia milii DCM Root Extract Encapsulated by Thermosensitive Immunoliposomes for Targeted Drug Delivery in Prostate Cancer Cells. Curr. Issues Mol. Biol..

[8] Babonaitė M., Striogaitė E., Grigorianaitė G., Lazutka J.R. (2024). In Vitro Evaluation of DNA Damage Induction by Silver (Ag), Gold (Au), Silica (SiO_2_), and Aluminum Oxide (Al_2_O_3_) Nanoparticles in Human Peripheral Blood Mononuclear Cells. Curr. Issues Mol. Biol..

[9] Tyavambiza C., Meyer M., Wusu A.D., Madiehe A., Meyer S. (2023). The Cytotoxicity of Cotyledon orbiculata Aqueous Extract and the Biogenic Silver Nanoparticles Derived from the Extract. Curr. Issues Mol. Biol..

[10] Morozova O., Isaeva E., Klinov D. (2023). Biodistribution of Fluorescent Albumin Nanoparticles among Organs of Laboratory Animals after Intranasal and Peroral Administration. Curr. Issues Mol. Biol..

[11] Matalqah S., Lafi Z., Asha S.Y. (2024). Hyaluronic Acid in Nanopharmaceuticals: An Overview. Curr. Issues Mol. Biol..

[12] Lukhele B.S., Bassey K., Witika B.A. (2023). The Utilization of Plant-Material-Loaded Vesicular Drug Delivery Systems in the Management of Pulmonary Diseases. Curr. Issues Mol. Biol..

